# Clinical Intervention Increases Rational Use of Proton Pump Inhibitors in the General Surgery Department

**DOI:** 10.3389/fphar.2022.864081

**Published:** 2022-04-25

**Authors:** Qiying Chen, Qiaohong Wang, Yin Zhang

**Affiliations:** ^1^ Department of Pharmacy, The Second Affiliated Hospital of Fujian Medical University, Quanzhou, China; ^2^ Department of Pharmacy, QuanZhou Women’s and Children’s Hospital, Quanzhou, China

**Keywords:** clinical pharmacist, proton pump inhibitors, PDCA cycle, pareto chart, costeffectiveness analysis

## Abstract

This study aimed to evaluate the role of the clinical pharmacist in the rational use of proton pump inhibitors (PPIs) in a general surgery department. All enrolled patients had attended the general surgery department of a tertiary hospital. This single-center prospective study compared differences in the overall rate of rational PPI use, proportion of unindicated PPI use, utilization rate, average defined daily dose (DDD), drug costs, PPI costs, and cost-effectiveness of clinical pharmacist intervention between the intervention (538 cases) and control (536 cases) groups. In the intervention group, Pareto and fishbone diagram analyses were combined with the Plan-Do-Check-Act cycle; Statistical Package for the Social Sciences was used for analyzing all data. The overall rate of rational PPI use was significantly higher in the intervention group than in the control group (*p* < 0.01). The proportion of unindicated PPI use, utilization rate, average DDD, drug costs, and PPI costs were significantly lower in the intervention group than in the control group (*p* < 0.05). Cost-effectiveness analysis for the overall rate of rational PPI use indicated a positive impact of intervention, with economic benefits in the intervention group. Clinical pharmacist intervention for rational use of PPIs in general surgery departments could significantly increase the overall rate of rational PPI use; it could also reduce the proportion of unindicated PPI use, utilization rates, average DDDs, drug costs, and PPIs costs. Pharmacist intervention also offers economic benefits by improving the overall rate of rational PPI use.

## 1 Introduction

Proton pump inhibitors (PPIs) are commonly used to inhibit gastric acid secretion. They bind irreversibly with the proton pump in gastric parietal cells and inactivate them, thereby blocking the last channel of gastric acid secretion; this has a strong inhibitory effect on gastric acid production. According to the Annual Report on Hospital Drug Use Monitoring of the Chinese Pharmaceutical Association, 2018, PPIs ranked 4th among the top 5 digestive system drugs (Chinese Pharmaceutical Association, 2019). Owing to their extensive clinical use, the irrational use of PPIs is widespread. A study on 96669 patients in the United States showed that 78–82% of PPI prescriptions are irrational (Leri et al., 2013). A 4-years study in South Korea also showed that 50% of patients used PPIs without indication (Shin, 2015). In China, the rates of rational PPI use are also not optimal, and the unindicated use of medication is common (Liu et al., 2019). In a previous study, we found that among the top ten drugs used at our hospital, PPIs were the third most common. In terms of prescription volumes, the general surgery department repeatedly featured among the top ten; data indicated the rate of irrational PPI use between June and November 2018 to be as high as 64.11%. Irrational drug use increases the incidence of adverse drug reactions (ADR), including short-term allergies, acute interstitial nephritis, and acute attacks of gout, among others. Long-term use increases the risks of vitamin B12 deficiency, fractures, pneumonia, and other conditions. A study by Ahrens et al. showed that insufficient awareness of the harmful effects of irrational PPI use among doctors leads to an increase in unindicated prescriptions (Ahrens et al., 2012). Therefore, it is essential to promote the rational use of PPIs to reduce ADR and improve the quality of medical care.

Clinical pharmacists can utilize their professional pharmaceutical knowledge to compensate for any deficiency of knowledge among clinicians; this will ensure the safety of patients and improve the quality of medical care. As per the Notice on Issuing Opinions on Strengthening the Administration of Pharmaceutical Affairs in Medical Institutions and Promoting Rational Use of Medicines (Chinese National Health Medical, 2020), clinical pharmacists are required to participate in clinical treatment, conduct medical order reviews, and provide medication education, among other activities. The multidisciplinary team process must involve clinical pharmacists; this affirms their role in promoting rational drug use. Some studies have shown that clinical pharmacists can promote safety and rational drug use through intervention in treatment and prescription reviews (Ferracini et al., 2011); they can also reduce the use and expenditure of PPIs and increase rates of rational PPI use (Luo et al., 2018). The construction of a clinical pharmaceutical care system among tertiary hospitals in China has achieved initial results (Cui et al., 2018). Our hospital has a clinical pharmacist training base approved by the National Health Commission; there are eight trained pharmacists, including two in the digestive field, who provide professional support for research.

In summary, although PPIs are widely used in clinical practice, physicians have insufficient knowledge on their rational use; this may increase both, the occurrence of ADR and the medical burden on patients. Although the general surgery department in our hospital uses PPIs effectively, the rate of rational use is not optimal. Therefore, this study evaluated whether pharmacist intervention could increase the rate of rational PPI use and reduce the medical burden on patients in the department of general surgery at our hospital. This study employed Pareto chart and fishbone diagram analyses combined with Plan-Do-Check-Act (PDCA) cycle management to explore the role of clinical pharmacists in promoting the rational use of PPIs in the general surgery department. It also aimed to provide an intervention model for other drug treatments and comprehensively evaluate the effectiveness of clinical pharmacist intervention in a general surgery department from the perspectives of PPI usage patterns, appropriateness, and safety.

## 2 Materials and Methods

### 2.1 Patient Selection

**FIGURE 1:**
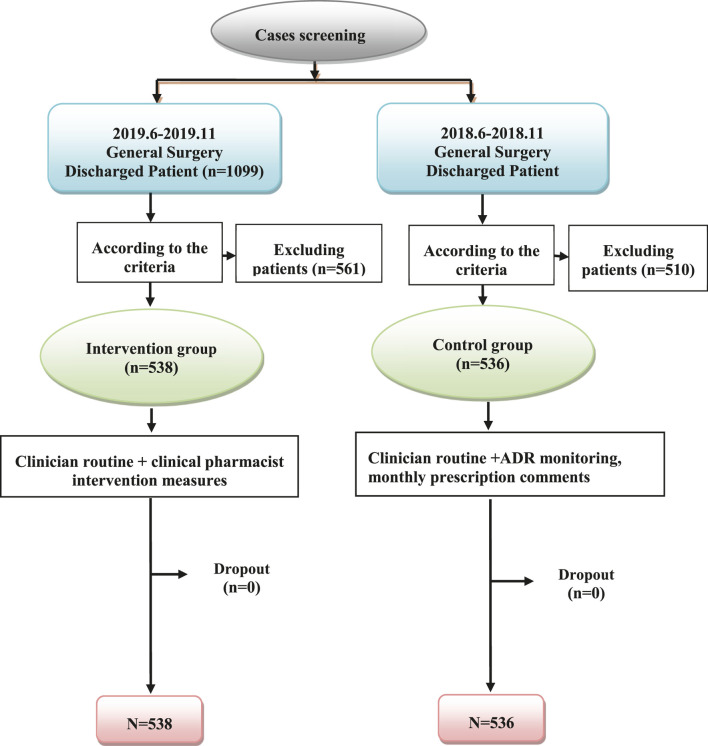
Flow chart of case screening.

All patients enrolled in this single-center prospective study had attended the Second Affiliated Hospital of the Fujian Medical University between June and November, 2018 (control group) and June and November 2019 (intervention group). The patients who were treated by the Hepatobiliary and Pancreatic, Gastrointestinal, and Thyroid and Breast Surgery groups (groups A, B, and C, respectively) of the Department of General Surgery, and who met the criteria for inclusion and exclusion were included. In the control group, patients were managed according to the clinicians’ routine diagnosis and treatment; in the intervention group, clinical pharmacists intervened during treatment following routine diagnosis. This trial was performed in compliance with international clinical practice guidelines, and in accordance with the principles of the Declaration of Helsinki routine diagnosis (Figure 1). The study was approved by the ethics committee of the hospital [project, approval number: (2019) Fuyifuer Ethnics Shenzi (231)]. This study was registered in the Chinese Clinical Trial Registry (registration number: ChiCTR1900025782).

The inclusion and exclusion criteria were developed by clinical pharmacists and experienced general surgeons. The criteria were developed based on the disease types of admitted patients, scope of diagnosis and treatment in the general surgery department, and the needs of this study. Patients who underwent treatment under groups A, B, and C of the Department of General Surgery between June and November, 2018 and between June and November, 2019 were included. The inclusion criteria were as follows:

a) A primary diagnosis related to any site relevant to general surgical intervention, including the esophagus, stomach, duodenum, colon, rectum, small intestine, appendix, hepatobiliary and pancreatic system, spleen, breast, and thyroid, among others; cases with abdominal hernia were also included.
b) Patients aged ≥18 years.
c) Cases where complete medical records data were available.

Exclusion Criteria

a) Transferred patients (including transfer-in and transfer-out cases)
b) Patients who had not been discharged during the sampling period of cases
c) Patients with a history of antineoplastic therapy
d) Patients not receiving medication
e) Patients with mental illness or cognitive impairment
f) Patients with a history of gastrointestinal ulcer

### 2.2 Sample Size Calculation

The sample size required for the comparison of two sample means and two sample rates was calculated using the following formula (Sun, 2010): $n=2{\left[\frac{\left(\mu \alpha +\mu \beta \right)\sigma }{\delta }\right]}^{2}+\frac{1}{4}\mu \alpha^{2}$ and $n=1641.4{\left[\frac{\left(u\alpha +u\beta \right)}{\sin^{-1}\sqrt{p1}-\sin^{-1}\sqrt{p2}}\right]}^{2}$ , where *n* was the sample size of each group, *σ* was the population standard deviation, *δ* = *μ*_1_-*μ*_2_ was the difference between the two population means, and *p*1 and *p*2 were the two population rates, respectively. The values *α* = 0.05, *u*0.05/2 = 1.96, *β* = 0.10, *u*0.10 = 1.28, *σ*, *δ*, and *p*_1_ and *p*_2_ were obtained by pre-experiments. The primary data of the pre-experiments included the PPIs utilization rate, overall rate of rational PPI use, and average defined daily doses (DDDs). Considering a PPI utilization rate of n ≈ 79.25, overall rational PPI use rate of n ≈ 49.16, and average PPI DDDs of n ≈ 452.78 in the primary data, the sample size required by the Wilcoxon rank-sum test was 1.053-fold that required by the *t* test (Page et al., 2013); this amounted to approximately 477 cases. The study was performed for 6 months based on the number of cases needed in the control and intervention groups, considering the various confounding factors in the process of the study and the three types of data from the pre-experiments; 536 and 538 cases were finally obtained in the control and intervention groups, respectively.

### 2.3 Intervention

The PPIs Review Guidelines of the Second Affiliated Hospital of Fujian Medical University (Supplementary Appendix A1 were considered as the evaluation standard for this study. Clinical pharmacists (authors of this study) conducted one cycle of interventions under the guidance of their senior colleagues.

The interventions involved the following steps:

1) In addition to assessment of actual cases, a lecture on the rational use of PPIs was provided to the medical staff every quarter.
2) Clinical pharmacists participated in ward rounds and suggested medications at any time point considering the following parameters: medication indications, drug selection, frequency of administration, course of treatment, and drug combinations in consideration of patient age and liver and kidney function. Patients were monitored individually.
3) Irrational PPI prescriptions were intercepted using rational drug use software. The interception rules are shown in Supplementary Appendix A2.
4) Clinical pharmacists conducted a prescription audit every morning, strengthened audit of indications, communicated and provided feedback to doctors over time, reviewed prescriptions on the afternoon of the same day, and reminded doctors who had not revised the prescriptions following communication.
5) The clinical pharmacy department enlisted all essential monitoring drugs and checked inpatient medical records every month; in addition, clinical pharmacists commented on, analyzed, and summarized the records. Clinicians were provided feedback on the results and offered rectification advice. The findings were made available on the hospital intranet.
6) Clinical pharmacists monitored the occurrence of PPI-related ADR, evaluated the relevance of ADR, and intervened as appropriate.
7) Clinical pharmacists communicated with clinicians who had multiple problem prescriptions, provided suggestions for improvement, and followed-up the revised prescriptions.
8) Clinical pharmacists summarized and submitted a list of the irrational prescriptions with explanations to the medical department every month; the departmental director was asked to provide written feedback, which was included in the comprehensive quality assessment system of the department. The pharmacy department reported to the financial department for deduction if irrational prescriptions were found more than twice a month.

### 2.4 PDCA Cycle, Pareto Diagram, and Fishbone Diagram

The PDCA cycle includes four stages: plan, do, check, and act; it has been widely used in rational drug use interventions (Wang et al., 2019; Zhong et al., 2020). The primary process is to solve a problem through one cycle, summarize, find new problems, and propose new goals to enter the next cycle step by step. The Pareto diagram is used to determine the key factors causing quality problems, and analyze the current situation and main factors contributing to irrational drug use; this can expedite analysis and enhance the intuition of trend analysis (Kulkarni et al., 2018). The fishbone diagram analyzes the causes of irrational drug use; this aids in intuitively discovering the problem to find effective improvement measures. In this study, the Pareto diagram was used to analyze the current situation of drug use. Combined with a fishbone diagram, it helped to determine the root cause of the situation; this has significant effect on interventions related to clinical medication (Li et al., 2018; Ji et al., 2020).

#### 2.4.1 Plan

The plan involved the following:

##### 2.4.1.1 Current Situation Investigation

**FIGURE 2:**
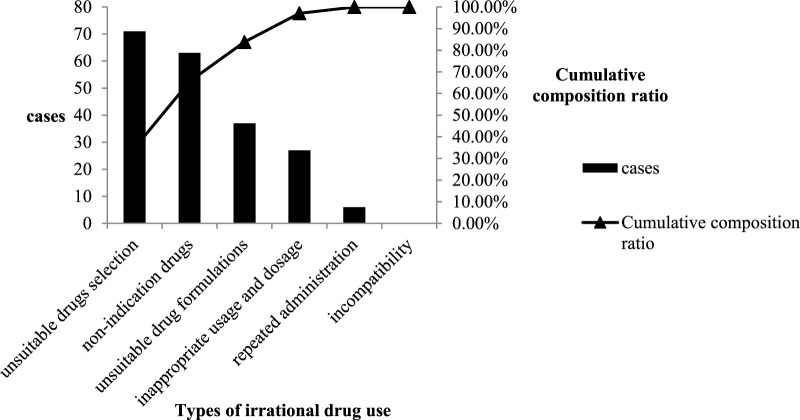
Pareto chart of types of irrational drug use in the control group.

**FIGURE 3:**
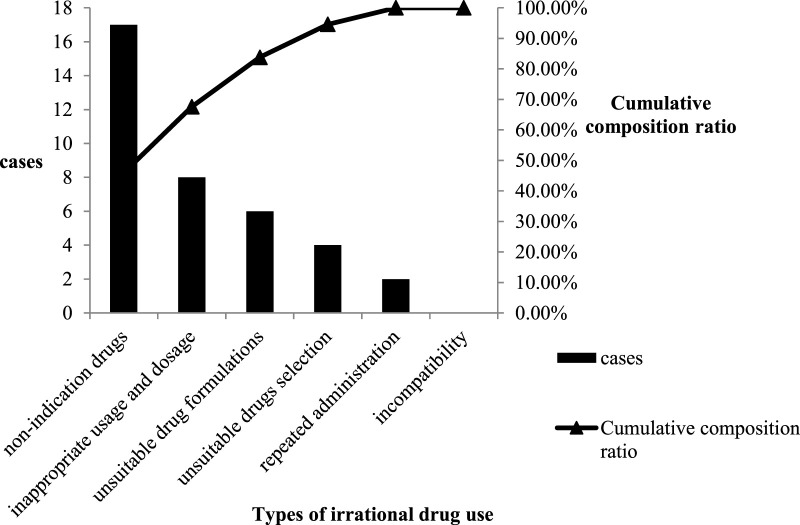
Pareto chart of types of irrational drug use in cycle.

The rationality in the control group and the first circulation cases were evaluated according to the evaluation criteria. The Pareto chart on the irrational drug use types was also drawn accordingly, as shown in Figures 2, 3. Among the 536 cases in the control group, the utilization and irrational drug use rates of PPIs were 53.5 and 64.11%, respectively; there were 204 irrational prescriptions of 6 types. The primary factor (type A) included unsuitable drug selection and use of unindicated drugs, and the second factor (type B) included use of unsuitable drug formulations; inappropriate use and dosage, repeated administration, and incompatibility constituted general factor type C.

##### 2.4.1.2 Cause Analysis

**FIGURE 4:**
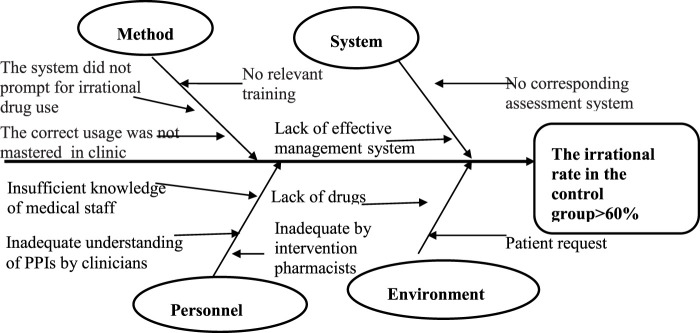
Fishbone diagram analysis for irrational PPI use in the control group.

The fishbone diagram (Figure 4) was used to identify the root causes of irrational PPI use. The four involved reasons include the system, method, personnel, and environment.

##### 2.4.1.3 Determining the Intervention Measures and Objectives

The intervention measures were formulated and intervention objectives were determined based on the primary factor type A, combined with the root causes of the current situation. This step mainly focused on identifying inappropriate or unindicated drug use and inappropriate usage and dosage, to improve the overall rate of rational drug use and reduce the utilization rate.

#### 2.4.2 Do

The clinical pharmacist interventions mentioned in Section 2.3 were implemented according to the intervention objectives. The table for utilization and rationality evaluation of PPIs in hospitalized patients (Schedule 1) was used as a tool to collect patients’ data.

#### 2.4.3 Check

The clinical pharmacist comprehensively analyzed the problems with PPI prescriptions and provided the clinicians with continuous feedback for improvement over time. The main problems related to rational drug use training were summarized every quarter. After implementing the rectification measures, the clinical pharmacists observed a gradual decline in the number cases receiving PPIs in the general surgery department.

#### 2.4.4 Action

Instances of irrational drug use were summarized and analyzed at each stage. The feasibility and effectiveness of the intervention measures in the previous stage were also evaluated simultaneously; this was performed in addition to determining the main objectives of the intervention in the next stage, summarizing and standardizing the practical measures, improving measures for rectification, and continuing rectification of unsolved problems in the next stage.

##### 2.4.4.1 Primary Endpoints

The main endpoints included the overall rate of rational PPI prescriptions, proportion of unindicated PPI use, utilization rate of PPIs, average DDDs of PPIs, drug costs, PPI costs, and cost-effectiveness analysis of clinical pharmacist intervention.

##### 2.4.4.2 Secondary Endpoints

The secondary endpoints included the distribution of rationality evaluation results, distribution of therapeutic and preventive drugs, and proportion of oral medication use.

Some indicators have been described in Supplementary Appendix A3.

### 2.5 Cost Measurement

This study performed cost estimation from the perspective of hospitals. The control group only needed to pay the average salary for the pharmacist. The monthly salary of pharmacists comprises 6 components:¥ 734.51, which is based on the post salary (¥ 1490) + level-based salary (¥ 472) + post allowance (¥ 335) + subsidy (¥ 1199) + performance (¥ 4500) - 5 insurances and 1 fund (¥ 1261.49) for junior pharmacists in Fujian public institutions The study lasted for 12 months, and the average annual salary of pharmacists was ¥ 6734.51 × 12 = ¥ 80814.12. The average annual cost of licensed training for each pharmacist is ¥ 400; the cost in the control group was therefore ¥ 80814.12 + ¥ 400 = ¥ 81214.12. The intervention group implements clinical pharmacist intervention. Assuming that the hourly rate of pharmaceutical service fee is equal to the hourly rate of pharmacist’s salary. The total time cost of clinical pharmacists for pharmaceutical intervention is shown in Table 1. The average cost of specialized clinical pharmacist training for each clinical pharmacist is ¥ 6000. Assuming that the training can serve for 30 years and the discount rate is 5% (Pharmacoeconomics Committee of Chinese Pharmaceutical Association, 2019), the annual cost is about ¥ 390.31 (Sun, 2014). The average annual training cost for clinical pharmacists is ¥ 1000, and the average annual training cost for licensed pharmacists is ¥ 400. In this study, the cost of the intervention group was calculated as follows: ① The average annual salary of pharmacists = ¥ 6734.51 × 12 = ¥ 80814.12; ② The time cost of clinical pharmacists in the intervention group = ¥ 18014.78; ③ The training cost of pharmacists in the intervention group = ¥ 390.31 + ¥ 1 000 + ¥ 400 = ¥ 1790.31. Therefore, in theory, the cost of PPIs intervention for clinical pharmacists in the hospital is the sum of the above three items (Li et al., 2017; Long et al., 2014), that is ¥ 100619.21.

**TABLE 1:** Measurement of intervention components.

Intervention items	Time	Total time
PowerPoint making	2.5 h	2.5 × 4 = 10 h*
Training for clinician	1h	1 × 4 = 4 h*
Daily ward round + prescription review	1.5 h	1.5 × 20×12 = 360 h*
77 patients were intervened	15 min	15 × 77 = 1155 min = 19.25 h
Total		
Total time	10 + 4+360 + 120+19.25 + 18 = 393.25 h	
Average hourly salary of pharmacists	6734.51÷21÷7 ≈ 45.81yuan*	
Total salary corresponding to time cost	393.25 × 45.81 ≈ 18014.78yuan	

*4 implies that the training was conducted for 4 times during the study; 20 implies pharmacy ward round + prescription review was performed for 20 days every month; 12 implies that the study lasted for 12 months; 21 implies that clinical pharmacists worked 21 days a month; and 7 implies working 7 h a day.

### 2.6 Statistical Analysis

All the case data were analyzed using SPSS 23.0 software. The data were evaluated using the Shapiro-Wilk test to check the normality of the sample and the Levene’s test to check the homogeneity of variance in the two samples. In cases where the two samples conformed to the normal distribution, the data were presented as the mean ± standard error (*x* ± *s*); the general t-test was used when the variance was uniform, whereas the approximate t-test was used when the variance was not uniform. In cases where the two samples were not normally distributed, the data were presented as the median (interquartile range) and the Mann -Whitney U test was used. The count data were presented as the frequency, rate, or constituent ratio, using the chi-square test; differences with *p* values of <0.05 were considered statistically significant.

## 3 Results

### 3.1 Baseline Data

The study finally included 1074 cases, including 536 in the control group (aged 14–88 years) and 538 in the intervention group (aged 14–93 years); the baseline data of patients were not statistically significant and comparable (*p* > 0.05) (Table 2).

**TABLE 2:** Comparison of basic conditions.

Items	Control Group	Intervention Group	Total	*p* Value
Age (years)	52 (41,63)	54 (42,66)		0.053
Gender (n)			*n* = 1074	0.177
Male	236	259		
Female	300	279		
Medical groups (n)			*n* = 1074	0.132
A	165	175		
B	120	142		
C	251	221		
Operation or not (n)			*n* = 1074	0.158
Yes	185	164		
No	351	374		
Types of diseases (n)			*n* = 1074	0.297
Pancreatic diseases	21	31		
Liver diseases	66	74		
Biliary diseases	151	173		
Splenic diseases	2	3		
Esophagus, stomach, and duodenum related diseases	43	27		
Colorectal and small bowel diseases	74	69		
Breast diseases	71	81		
Thyroid diseases	36	34		
Abdominal hernia	29	17		
Appendicitis	43	29		
Drug combinations (n)			*n* = 1074	0.462
Non-steroidal anti-inflammatory drugs	327	389		
Glucocorticoids	119	122		
Anticoagulant and antiplatelet drugs	30	32		
Antibiotics	329	328		
Digestive system related drugs	362	363		
Discharge with PPIs (n)			*n* = 1074	0.175
Yes	81	66		
No	455	472		
Average length of stay (days)	7.00 (5,12)	7.00 (5,12)		0.272

### 3.2 Comparison of the Rationality of PPI Use

In the control group, PPIs were used in 287 cases, and the overall rate of rational use was 35.89%. In the intervention group, PPIs were used in 206 cases, and the overall rate of rational use was 68.45% (Table 3).

**TABLE 3:** Comparison of overall rational PPI use rate and medication purposes.

Group	Overall rate of rational PPI use		Medication indications		Medication indications n (%)	
	Reasonable cases	Unreasonable cases	Indicated medication	Unindicated medication	Therapeutic medication	Preventive medication
Control Group	103 (35.89%)	184 (64.11%)	229 (79.8%)	58 (20.2%)	104 (45.4%)	125 (54.6%)
Intervention Group	141 (68.45%)	65 (31.55%)	181 (88.8%)	25 (11.2%)	97 (53.6%)	84 (46.4%)
*p* value	<0.001		0.018		0.100	

**FIGURE 5:**
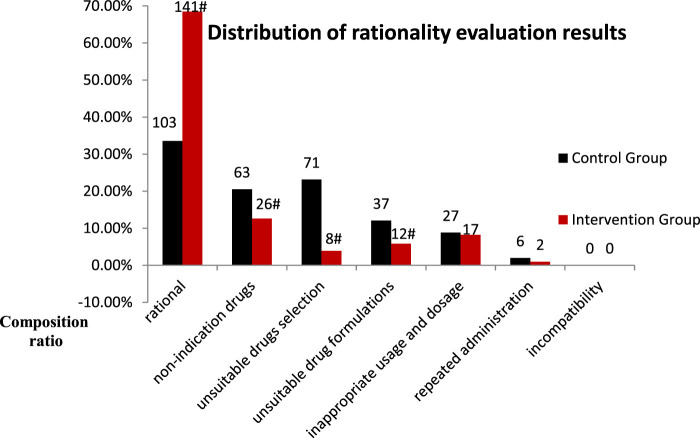
Distribution of rationality evaluation results for PPI use in the control and intervention groups (Compared with the control group. ^#^ *p* < 0.05).

The rationality evaluation results were categorized as follows: rational PPI use, unindicated drug use, unsuitable drug selection, unsuitable drug formulation, inappropriate usage and dosage, repeated administration, and incompatibility. The composition ratios of unindicated drugs, unsuitable drug selection, and unsuitable drug formulations were significantly lower in the intervention group than in the control group (*p <* 0.05) (Figure 5).

### 3.3 Cost-Effectiveness Analysis

#### 3.3.1 Comparison of PPI Use and Medical Expenses

There were no changes in PPI use or price adjustments during the study period. The utilization rate of PPIs, average DDDs of PPIs, proportion of intravenous drugs used, drug costs, and PPI costs were significantly lower in the intervention group than in the control group (Table 4).

**TABLE 4:** Comparisons of PPI use and medical expenses.

Items	Control Group (n = 536)	Intervention Group (n = 538)	*p* Value
PPI usage			
PPIs used	287 (53.5%)	206 (38.3%)	<0.001
PPIs not used	249 (46.5%)	332 (61.7%)	
Average DDDs of PPIs	5.30 ± 0.398	4.16 ± 0.369	<0.001
Oral DDDs (%)	705 (24.84%)	771.5 (34.51%)	<0.001
Intravenous DDDs	2133 (75.16%)	1464 (65.49%)	
Drug costs (in ¥)	3047.22 (1715.02,6635.11)	2517.78 (1399.92,6056.95)	0.036
PPI costs (in ¥)	77.78 (0,382.96)	0.00 (0,186.92)	<0.001

#### 3.3.2 Effect Measurement

As shown in Table 3, the overall rates of rational PPI use in the control and intervention groups were 35.89 and 68.45%, respectively.

#### 3.3.3 Cost-effectiveness Analysis of clinical pharmacist Intervention

In this study, the cost-effectiveness ratio of the lower-cost option (control group) was used as the threshold of willingness to pay. In terms of improving the overall rate of rational PPI use from the perspective of the hospital, the incremental cost-effectiveness ratio of the intervention/control group (ΔC/ΔE = 595.98 ¥/%) was lower than that of the control group (C/E = 2262.86) ¥/%). This finding indicated intervention to be more economical (Table 5).

**TABLE 5:** Comparison of costs and effects of clinical pharmacist intervention between control and intervention groups.

Group	Cost C (¥)	Effect E (%)	C/E (¥/%)	ΔC/ΔE (¥/%)
Control Group	81 214.12	35.89	2 262.86	595.38
Intervention Group	100 619.21	68.45	1 469.97	

#### 3.3.4 Sensitivity Analysis

Sensitivity analysis showed that the findings of this study tended to be stable when the hourly salary of the pharmacists changed by up to 50%. This implied that under different hourly wage levels, the economic efficiency of the intervention group in improving the overall rate of rational PPI use was better than that of the control group. The findings showed that the lower the hourly wage, the greater was the difference between the incremental ratio and the C1/E in the control group (Table 6).

**TABLE 6:** Results of sensitivity analysis for hourly salary changes among clinical pharmacists.

Range of hourly wage change	Control Group	Intervention Group	ControlGroup	Intervention Group	ΔC/ΔE (¥/%)
	C_1_ (¥)	C_2_ (¥)	C_1_/E (¥/%)	C_2_/E (¥/%)	
0(%)	81 214.12	100 619.21	2 262.86	1 469.97	595.98
2(%)0	81 214.12	104 222.17	2 262.86	1 522.60	706.64
40(%)	81 214.12	107 825.13	2 262.86	1 575.24	817.29
50(%)	81 214.12	109 626.60	2 262.86	1 601.56	872.62

(C_1_, cost in the control group; C_2_, cost in the intervention group; E, overall rate of rational PPI, use/%).

## 4 Discussion

In clinical practice, many medication-related problems are commonly ignored by doctors. The optimization of issues related to the selection, administration, usage, and dosage of drugs, may lead to the achievement of better therapeutic effects. Clinical pharmacists should therefore provide professional expertise, take initiatives to find clinical medication-related problems during daily ward rounds, analyze causes of the problems, and formulate appropriate medication plans. The collaboration between doctors and clinical pharmacists is essential for the development of pharmaceutical care. In this context, the provision of smooth hospital drug supply channels, timely supply, timely coordination, routine rational drug use, case discussion, and academic exchange can increase collaboration between doctors and clinical pharmacists.

The findings from this study suggest that intervention by clinical pharmacists significantly improved the overall rate of rational PPI use in the general surgery department, with a significant reduction in the proportion of unindicated drug usage (Table 3). This indicates that continuous intervention by clinical pharmacists can provide clinicians with better understanding on the indications of PPI use, thereby reducing their abuse. Comparison of the results of rationality evaluation before and after intervention indicated that clinical pharmacist intervention had significant impact on unindicated drug use, unsuitable drug selection, and use of unsuitable drug formulations (Figure 3). In particular, the finding of significant improvement in drug selection was more aligned with our hospital policy regarding omeprazole prescriptions. The policy considers omeprazole to be the only injection for preventive purposes, as only omeprazole (Losec) injection is clearly mentioned in the instructions for preventive drugs. Studies from the literature also indicate that intravenous pantoprazole and omeprazole are equivalent for the prevention of stress ulcers (Messori et al., 2014). The significant improvement in the use of unsuitable drug formulations aligns with our hospital policy of oral usage without intravenous infusions. Studies have shown have no statistical difference in basal and maximal acid production between patients with gastroesophageal reflux disease receiving either intravenous or oral esomeprazole (Metz et al., 2005). A meta-analysis also showed that oral PPIs are viable and safe alternatives to intravenous PPIs for patients with bleeding peptic ulcers, and may become the first choice for treatment in future (Jian et al., 2016). In our study, no incompatibility was found in either group before and after the intervention (Figure 3). This indicates that additional use of rational drug use software can help doctors and nurses better understand the compatibility of injecs PPIs. In this context, injectable PPIs are mostly weakly alkaline; mixed intravenous infusions with other acidic drugs (such as amino toluene acid, ethyl phenol sulfonate, and vitamin B6, among others) should therefore be avoided to reduce ADR (He and Fu, 2017). The findings from this study suggested a significant reduction in drug and PPI related costs following intervention (Table 4). These findings are consistent with those observed by Xin et al.; they also found that pharmacist intervention regarding rational PPI use can reduce total drug and PPI related costs Xin et al. (2018). Previous studies on rationality of drug use by clinical pharmacists have mostly adopted parallel comparisons between control and intervention groups. In this study, a cost-effectiveness analysis was added in order to provide a reference for calculation of pharmaceutical service costs among medical institutions in China. The results showed that when the average hourly salary of pharmacists was set at ¥ 45.81/hour, the hospital would need to invest ¥ 595.38 more for every 1% increase in the overall rationality rate of PPI use (Table 6); this theoretically caused an increase in hospital payment costs. However, clinical pharmacist intervention regarding the rational use of PPIs can improve the level of rational drug use among clinicians, reduce the incidence of ADR, and improve the quality of medical treatment. From the patients’ perspective, a reduction in unnecessary medical expenses helps improve patient satisfaction with the hospital. From the hospital perspective, improving the rate of rational PPI use and reducing unnecessary PPI-related expenses help save costs related to medical insurance. All of these factors have a positive impact on the economic and social benefits of the hospital. This real-world study highlights the role and significance of clinical pharmacists in rational drug use. Clinical pharmacist intervention is helpful for improving awareness on rational drug use and medication-related behaviors, that may help prevent and treat diseases effectively, safely, and economically. This successful strategy can therefore be applied to the rational use of other types of drugs or departments in hospitals.

In this single-center prospective research performed at the general surgery department of our hospital, clinical pharmacists participated in the entire process. Pareto and fishbone diagram analyses were combined with the PDCA cycle for management of the pharmacist intervention process, to explore the role of clinical pharmacists in the rational use of PPIs. Owing to the limited study period, the pharmacist intervention measures were mainly aimed at clinicians; however, the effect indicators of pharmacist intervention need further improvement. Future research is expected to improve clinical pharmacist intervention measures; it may also expand the spectrum of clinical pharmaceutical care from more levels to help clinical pharmacists serve patients better. Research is needed on increasing recognition of the role of clinical pharmacists among clinicians and patients, and promoting rational use of clinical medication.

In conclusion, through continuous intervention regarding rational PPI use in the general surgery department, clinical pharmacists used Pareto fishbone diagram analyses combined with the PDCA cycle management intervention process to promote continuous optimization of intervention. It is essential that physicians generally recognize the role of clinical pharmacists, to ensure that the latter gradually become essential members of the treatment team; this will help serve patients better and promote rational use of clinical medication.

## Data Availability

The original contributions presented in the study are included in the article/Supplementary Material, further inquiries can be directed to the corresponding author.
