# Supplementary material for: Clinical Intervention Increases Rational Use of Proton Pump Inhibitors in the General Surgery Department
Source: Front Pharmacol. 2022 Apr 25;13:864081. doi: 10.3389/fphar.2022.864081 (PMC9083545; doi:10.3389/fphar.2022.864081)
Supplement: Supplementary file 1 [file DataSheet1.docx]

**Appendix**

**Schedule 1 Table for utilization and rationality evaluation of proton pump inhibitors in hospitalized patients**

**Department Bed No:**

| Name |  | Gender | |  | | Age | |  | | Medical record No | |  | |
| --- | --- | --- | --- | --- | --- | --- | --- | --- | --- | --- | --- | --- | --- |
| Hospitalization expenses /yuan | | |  | | Drug cost/yuan | | | |  | | PPIs cost/yuan | |  |
| Admission date Discharge date Total days _____ | | | | | | | | | | | | | |
| Main diagnosis | | |  | | | | | | | | | | |
| Operative name  （Operation grade /Date） | |  | | | | | | | | | Fasting or not |  | |
| **Past medical history and medication history：** | | | | | | | | | | | | | |
| **History of drug allergy：** | | | | | | | | | | | | | |
| **Laboratory index（Related to PPIs）**   | **Date** |  | | --- | --- | |  |  | |  |  | |  |  | | | | | | | | | | | | | | |
| **Gastrointestinal discomfort or abnormal symptoms** | | |  | | | | | | | | | | |
| **Digestive system examination results** | | |  | | | | | | | | | | |
| | **Drug use (PPI+combination medication)** | | | | --- | --- | --- | | Drug | Specific medical order | | |  |  | | |  |  | | |  |  | | | Incompatibility and interaction | |  | | | | | | | | | | | | | | |
| **PPI medication purpose:**1 Preventive 2. Therapeutic 3. No purpose  **Therapeutic indications：**1. Peptic ulcer 2. Eradication of Helicobacter pylori 3. Gastroesophageal reflux disease 4. Abnormal gastric acid secretion 5. Upper gastrointestinal bleeding 6. Chronic gastritis 7. Epigastric pain syndrome 8. Pancreatitis  **PPI medication purpose：__________；indications：** | | | | | | | | | | | | | |
| **Preventive medication indications：**1. NSAIDs related gastroduodenal mucosal injury 2. Glucocorticoid related gastroduodenal mucosal injury： 3. Antiplatelet drugs related gastroduodenal mucosal injury 4. Preventive use of PPI during perioperative period 5. Risk factors for stress ulcer(SU)  **indications：______**  Risk factors for stress ulcer**：**  PPIs should be used to prevent SU with one of the following high risk factors：  a. Mechanical ventilation ＞ 48 hours;  b. Coagulation mechanism disorder（INR＞1.5，platelet＜50×10^9^/L or Partial prothrombin time＞2 times）;  c. History of peptic ulcer or upper gastrointestinal bleeding within 1 year;  d. Severe head injury and cervical spinal cord injury, Clasgrow Coma Score≤10 points（Or cannot execute simple commands）;  e. Severe burns（Burn area＞30%）;  f. Severe trauma and multiple injuries;  g. Various difficult and complicated operations（Operation time> 3 hours, operation grade ≥ grade 3）;  h. Kidney dysfunction or liver dysfunction（Including severe obstructive jaundice）;  i. ARDS;  j. Shock or persistent low blood pressure（persistent low blood pressure＞30 min, Shock refers to systolic blood pressure <90 mmHg or lower than basal blood pressure>40 mmHg）;  k. Sepsis;  l. Cardiovascular accident;  m. Severe psychological stress, such as mental trauma, excessive stress, etc.。  Or when you have both of the following risk factors  o. ICU hospital stay> 1 week；  p. Duration of fecal occult blood > 3 days； | | | | | | | | | | | | | |
| **Evaluation of the rationality of PPI medication：**   | **Drug** | **Is it reasonable** | | **Indications** | **Drug selection** | **drug formulations** | **Usage and dosage** | **Repeated administration** | **Incompatibility** | | --- | --- | --- | --- | --- | --- | --- | --- | --- | |  |  | |  |  |  |  |  |  | |  |  | |  |  |  |  |  |  | |  |  | |  |  |  |  |  |  | | **Overall evaluation results** | |  | | | | | | | | **Note: unreasonably write down the specific reasons, if one item is unreasonable, it is overall unreasonable** | | | | | | | | | | | | | | | | | | | | | | |
| **Occurrence and treatment of adverse reactions** | | |  | | | | | | | | | | |
| Does stress ulcer occur | | |  | | | | Hospital acquired pneumonia | | | |  | | |

**Appendix 1 Proton Pump Inhibitors (PPIs) Review Guidelines of** **The Second Affiliated Hospital of Fujian Medical University**

Note: the guideline was formulated with reference to the current domestic guidelines and consensus and in combination with the actual situation of our hospital. It has been publicized on the hospital's intranet and officially implemented in April 2018 without objection.

|  | Table 1 Use of standard dose PPIs | | | |
| --- | --- | --- | --- | --- |
| Route of administration | | Drug | Standard dose | Frequency |
|  | Injection | Omeprazole injection | 40mg | qd |
|  |  | Pantoprazole sodium lyophilized powder | 40mg | qd |
|  |  | Lansoprazole lyophilized powder | 30mg | qd |
|  |  | Esomeprazole Sodium Lyophilized Powder | 40mg | qd |
|  |  | Rabeprazole sodium lyophilized powder | 20mg | qd |
|  | Oral medication | Omeprazole Enteric-coated Tablets | 20mg | qd |
|  |  | Lansoprazole capsule | 30mg | qd |
|  |  | Rabeprazole sodium enteric coated tablets | 10mg | qd |
|  |  | Esomeprazole enteric capsule | 20mg | qd |
|  |  | Esomeprazole magnesium enteric coated tablets | 20mg | qd |
|  |  | Pantoprazole Enteric-Coated Tablets | 40mg | qd |
|  |  | Iprazole enteric coated tablets | 10mg | qd |

**1. Therapeutic medication**

**（1）Peptic ulcer:** Including gastric ulcer, duodenal ulcer, artificial ulcer caused by iatrogenic or physical and chemical factor. Peptic ulcer is usually treated with standard dose of PPIs (see Table 1), 1-2 times a day. Take medicine half an hour before breakfast. It can be administered intravenously under fasting. It is recommended to stop the drug when oral feeding can meet the required nutrition.

**（2）Combined with antibiotics to eradicate Helicobacter pylori:** PPIs mainly enhances the sensitivity of antibiotics by increasing the pH in the stomach. When PPIs are used to eradicate HP, double standard doses are usually used, twice a day, and the course of treatment is 10 to 14 days.

**（3）Gastroesophageal reflux disease(GERD):** Standard dose of PPIs, 1-2 times a day. If the treatment is ineffective, double doses can be used. When double-dose PPIs are used, they should be taken twice before breakfast and before dinner respectively. The maintenance dose is the standard dose or halved.

**（4）Abnormal gastric acid secretion due to abnormal increase in gastrin secretion**: Such as Zollinger-Ellison syndrome, gastrinoma and so on. Usually double the standard dose of PPIs is used twice a day. If the basal gastric acid secretion (BAO) is greater than 10 mmol/h, the dose needs to be increased to achieve the desired acid suppression effect.

**（5）Upper gastrointestinal bleeding caused by various reasons:** It is recommended to use PPIs injections such as omeprazole or esomeprazole. When severe bleeding occurs, the "80+8" regimen can be used, that is, the first 80 mg intravenous bolus, the next 8 mg/h for 72 hours, and then the standard Dose of PPIs intravenous infusion, 2 times a day (omeprazole 40 mg, q12 h; pantoprazole 40 mg, q12 h; esomeprazole 40 mg, q12 h; lansoprazole 30 mg, q12 h ; Rabeprazole 20 mg, q12h), 3 to 5 days, after that oral standard dose of PPIs until the ulcer healed.

**（6）Chronic gastritis:** The standard dose of PPIs (Table 1) is usually used, once a day, half an hour before breakfast.

**（7）Upper abdominal pain syndrome in functional dyspepsia:** The standard dose of PPIs (Table 1) is usually used, once a day, half an hour before breakfast.

**（8）Pancreatitis**

①Indications for use: severe pancreatitis, acute pancreatitis with or without complications, chronic pancreatitis

②Drug selection:

a. For severe pancreatitis, omeprazole 80 mg qd or 40 mg, q12 h, pantoprazole 80 mg, qd or 40 mg, q12 h, esomeprazole 40 mg, Q12 h can be used.

　　b. Acute pancreatitis with or without complications and chronic pancreatitis shall be treated with omeprazole, pantoprazole, esomeprazole and other drugs according to the routine. Refer to the instructions for drug dosage: omeprazole 40 mg, 1 ~ 2 times a day; Pantoprazole 40 ~ 80 mg, 1 ~ 2 times a day; Esomeprazole 20 ~ 40 mg, qd.

③Course of treatment: the course of treatment should be based on the specific situation of the patient. Clinically, PPIs should be stopped if the patient has no abdominal pain and normal blood routine and amylase.

**2. Preventive medication: mainly to prevent the occurrence of stress ulcer (Su) or acute gastric mucosal injury.**

**（1）Non steroidal anti-inflammatory drugs(NSAIDs) related gastroduodenal mucosal injury:**

①Indications for use: patients used NSAIDs, if there is one of the following situations, PPIs is recommended to prevent gastrointestinal mucosal injury:

a. Patients with a history of peptic ulcer,

b. Dyspepsia or gastroesophageal reflux symptoms,

c. Patients receiving dual antiplatelet therapy,

d. Patients taking warfarin and other anticoagulant drugs at the same time,

e. Patients taking another NSAIDs (including low-dose or high-dose aspirin)

f. Patients with glucocorticoids

g. The patient was advised to eradicate Helicobacter pylori

h. Age > 65

i. Length of stay in ICU > 1 week

j. Duration of fecal occult blood > 3 days

②Drug selection: Omeprazole 20 ~ 40 mg, qd; Pantoprazole 40 mg, qd; Lansoprazole 30 mg, qd; Rabeprazole 10 ~ 20 mg, qd; Esomeprazole 20 ~ 40 mg, qd, iprazole 5 ~ 10 mg, qd. Oral administration is preferred. Intravenous administration is considered for those who cannot take oral administration. Omeprazole injection (Losec) can only be selected for intravenous use. The course of treatment depends on the course of NSAIDs used.

**（2）Glucocorticoid related gastroduodenal mucosal injury:**

①Indications for use: Patients use glucocorticoids. If there is one of the following factors, PPIs is recommended to prevent upper gastrointestinal mucosal injury：

a. PPIs can be used prophylactically for people whose dosage (taking prednisone as an example) is greater than 0.5 mg / (kg • d) (based on 60kg of patients, prednisone 30mg / dexamethasone 4.5mg / methylprednisolone 24mg per day);

b. At the same time using non-steroidal anti-inflammatory drugs, no matter what dose of glucocorticoid, PPIs should be given to prevent gastric mucosal damage;

c. Those with a history of peptic ulcer or bleeding within 1 year, regardless of the dose of glucocorticoid, should be given PPIs to prevent gastric mucosal damage;

d. For the long-term maintenance dose: 2.5~15.0 mg/d, the symptoms of gastrointestinal bleeding should be closely monitored, and PPIs should be given if necessary.

e. Length of stay in ICU > 1 week

f. Duration of fecal occult blood > 3 days

②Drug selection: Omeprazole 20 ~ 40 mg, qd or bid; Pantoprazole 40 mg, qd or bid; Lansoprazole 30 mg, qd or bid; Rabeprazole 10 ~ 20 mg, qd or bid; Esomeprazole 20 ~ 40 mg, qd or bid, iprazole 5 or 10mg, qd. Oral administration is preferred. Intravenous administration is considered only for those who cannot take oral administration. Omeprazole injection (Losec) can only be used for intravenous administration. The course of treatment depends on the course of treatment of glucocorticoids used.

**（3）抗血小板药物相关的应激性溃疡的防治**

①Indications for use: In order to minimize the digestive tract damage caused by antiplatelet therapy, it is recommended that clinicians adopt a standardized process for risk assessment and screening (Figure 1).

No

Yes

Assess indications for antiplatelet therapy

Assess the risk of gastrointestinal bleeding (in accordance with the following ≥1 items):

- History of peptic ulcer and complications
- History of gastrointestinal bleeding
- Dual antiplatelet therapy or combined anticoagulation therapy

The following ≥ two risk factors:

- Age > 65
- Use of glucocorticoids
- Indigestion or GERD

Preventive use of PPI or H2 receptor antagonist

Test for HP and give treatment if it is positive

Figure 1 　The treatment process to reduce the digestive tract injury in patients with antiplatelet therapy

②Drug selection: Omeprazole 20 ~ 40 mg, qd; Pantoprazole 40 mg, qd; Lansoprazole 30 mg, qd; Rabeprazole 10 ~ 20 mg, qd; Esomeprazole 20 ~ 40 mg, qd, iprazole 5 ~ 10 mg, qd. Oral administration is preferred. Intravenous administration is considered for those who cannot take oral administration. Omeprazole injection (Losec) can only be selected for intravenous use. Since clopidogrel may interact with omeprazole and esomeprazole, PPIs with less interaction such as rabeprazole, pantoprazole and iprazole are recommended when using clopidogrel. It is suggested that the time of combined application of PPIs should be determined according to the specific situation of patients. High risk patients can use PPIs in combination 6 months before antiplatelet drug treatment, and change to h2-ra or take PPIs intermittently after 6 months.

**（4）Guidelines for the preventive use of proton pump inhibitors during the perioperative period.**

a. When preventing postoperative Su before major surgery (surgery classified as grade III or above), PPIs for injection is not recommended. For patients who are planning to undergo major surgery, it is estimated that patients with postoperative SU may take acid inhibitors or antacids orally within one week before surgery to increase the pH of the stomach. Drug selection: Omeprazole 20 ~ 40 mg, qd; Pantoprazole 40 mg, qd; Lansoprazole 30 mg, qd; Rabeprazole 20 mg，qd；Esomeprazole 20 ~ 40 mg, qd, iprazole 5 ~ 10 mg, qd. Oral administration is preferred. Intravenous administration should be considered only for those who cannot take orally, and Omeprazole injection (Losec) can only be selected for intravenous use .

b. After major surgery, PPIs for injection can be given. Omeprazole injection (Losec) 40 mg, Q12 h, can be used continuously for 3 days. When the patient's condition is stable, can tolerate enteral nutrition or has eaten, and the clinical symptoms begin to improve, it is not recommended to continue to use PPIs for injection, but should be given orally. The occurrence of Su is mostly concentrated within 3 ~ 5 days of the primary disease, and a few can be extended to 2 weeks. Encourage early eating to neutralize gastric acid and enhance gastric mucosal barrier function to prevent su.

c. After general surgery (surgery classified as grade I and grade II), it is not a high-risk factor to prevent Su, and it is not recommended to use PPIs for injection after surgery.Prophylactic use of PPIs for injection is recommended only for patients in high-risk groups with Su

d. For patients with general liver disease (no severe jaundice, no coagulation disorders, no liver and kidney failure, etc.), it is not a high-risk factor for the prevention of SU, and it is not recommended to use PPIs for injection prophylactically after surgery.

**（6）Patients with high risk factors for stress ulcers need to use PPIs to prevent acute gastric mucosal injury**

①Indications for use：

PPIs should be used to prevent SU with one of the following high risk factors：

a. Mechanical ventilation ＞ 48 hours;

b. Coagulation mechanism disorder（INR＞1.5，platelet＜50×10^9^/L or Partial prothrombin time＞2 times）;

c. History of peptic ulcer or upper gastrointestinal bleeding within 1 year;

d. Severe head injury and cervical spinal cord injury, Clasgrow Coma Score≤10 points（Or cannot execute simple commands）;

e. Severe burns（Burn area＞30%）;

f. Severe trauma and multiple injuries;

g. Various difficult and complicated operations（Operation time> 3 hours, operation grade ≥ grade 3）;

h. Kidney dysfunction or liver dysfunction（Including severe obstructive jaundice）;

i. ARDS;

j. Shock or persistent low blood pressure（persistent low blood pressure＞30 min, Shock refers to systolic blood pressure <90 mmHg or lower than basal blood pressure>40 mmHg）;

k. Sepsis;

l. Cardiovascular accident;

m. Severe psychological stress, such as mental trauma, excessive stress, etc.。

Or when you have both of the following risk factors

o. ICU hospital stay> 1 week；

p. Duration of fecal occult blood > 3 days；

②Drug selection: It is recommended to give omeprazole injection (Losec) 40 mg, q12h, intravenous drip for 3 days after the emergence of high-risk factors.

　　③Indications for discontinuation：

It is recommended that patients have a reduced risk of clinical bleeding, can tolerate enteral nutrition or have eaten, clinical symptoms have begun to improve, or transferred to a general ward as the time to prevent SU drug withdrawal. However, for patients with high acid secretion (such as head surgery, severe burns), it is recommended to stop the drug until the oral intake can meet the required nutrients.

④For patients undergoing total gastrectomy, there is no indication for the use of PPIs.

**Appendix 2 Blocking reminder rules of reasonable medication software**

①ncompatibility and unsuitable solvent: PPIs for injection should be 0.9% sodium chloride injection, avoid mixed intravenous drip with other drugs, If it does not meet the requirements, it shall be intercepted.

②Route of administration: PPIs for injection should be administered by intravenous drip or micro-pump injection; PPIs for injection are only used for patients who are not suitable for oral medication. When prescribing PPIs for injection, a pop-up reminder should be given.

③Medication contraindications: the patient has a history of PPI or benzimidazole allergy, interception will be performed when PPIs are prescribed.

④Usage and dosage: The frequency of PPIs administration is 1 ~ 2 times a day. If it is more than 2 times, it will be remindedPPIs. The dosage of omeprazole, esmprazole and pantoprazole for injection shall not exceed 80 mg each time, and the dosage of lansoprazole and rabeprazole shall be 30 mg and 20 mg respectively. If the above dosage does not meet the requirements, it shall be reminded.

⑤Drug-drug interaction: The combination of PPIs could significantly reduce the bioavailability of gefitinib and azanavir; PPIs significantly reduced the absorption of posaconazole, erlotinib, ketoconazole and itraconazole; Omeprazole combined with clopidogrel, voriconazole or high-dose methotrexate are not advised; If the above combined medication occurs, pop-up reminder will be given.

**Appendix 3 Description of some indicators involved in this study**

**The overall rational rate of PPIs** There can be multiple PPI orders for patients during hospitalization or discharge with medication. According to the evaluation criteria, if one order is unreasonable in drug suitability, the overall evaluation is unreasonable. If all PPIs orders of the patient are reasonable, the overall evaluation is reasonable. Overall reasonable rate of PPIs = (number of cases evaluated as reasonable / total number of cases in each group) × 100%。

**The utilization rate of PPIs** Patients who have used PPIs during hospitalization or discharge with medication, the number of PPIs used is recorded as 1, PPIs utilization rate = (number of cases using PPIs in each group/total number of cases in each group) × 100%.

**The average DDDs of PPIs** According to to WHO ATC/DDD Index 2019, "*Pharmacopeia of the People's Republic of China•Clinical Medication Instructions*" (2015 edition), "*Chinese Pharmacist and Physician Clinical Medication Guide*", "*New Pharmacology*" (17 edition), average daily dose in drug instructions, etc., determine the defined daily dose (DDD) of each drug, and calculate defined daily doses ( DDDs). DDDs = total drug consumption / DDD. DDDs is additive. DDDs of similar drugs can be added together. The more the DDDs value, the higher the use frequency of this drug; Average DDDs of drugs = sum of DDDs of all PPIs for patients in each group / total number of patients in each group. See Appendix 5 for PPIs drug DDD involved in this study.

**Purpose of medication** The purpose of medication includes medication without indication, therapeutic medication and preventive medication. If the patient has both therapeutic and preventive purposes, they are classified as therapeutic drugs.

Therapeutic drugs include PPIs used for the treatment of peptic ulcer, eradication of Helicobacter pylori, gastroesophageal reflux disease, abnormal gastric acid secretion, upper gastrointestinal bleeding, chronic gastritis, upper abdominal pain syndrome and pancreatitis; Preventive medication includes PPIs used for the prevention and treatment of non steroidal anti-inflammatory drugs (NSAIDs) related gastroduodenal mucosal injury, glucocorticoid related gastroduodenal mucosal injury, antiplatelet drug related stress ulcer (Su), perioperative preventive drugs, and the prevention of SU high-risk factors.

**Rationality evaluation results** According to the suitability classification of prescription review results in the "*Hospital Prescription Review Management Regulations (Trial) 2010 Edition*", the rationality evaluation results are divided into: reasonable and unreasonable. Unreasonable types include non-indication drugs, unsuitable drugs selection, unsuitable drug formulation, inappropriate usage and dosage, incompatibility and repeated administration.

The unsuitable drugs selection includes the use of injections other than omeprazole for preventive medication without special circumstances, and the selection of PPIs that can lead to DDI due to enzymatic metabolism of liver drugs (such as omeprazole, lansoprazole and esomeprazole).The unsuitable drug is that patients who can take drugs orally choose injections.The inappropriate usage and dosage includes medication that exceeds the course or dose recommended in the review guidelines. Incompatibility shows that the injection uses weak acidic solvent (such as glucose injection) or is mixed with other acidic drugs (such as aminotoluene acid, ethylphensulfoamine, vitamin B6, etc.); Repeated administration is manifested by prescribing two antacids at the same time, such as PPI and H2 receptor inhibitors.

**Cost-Effectiveness Analysis** Cost-Effectiveness Analysis (CEA) was used to analyze before and after the intervention from the perspective of the hospital.

（1）**Cost(C)** The difference between the control group and the intervention group in this study is that the intervention group has undergone clinical pharmacist intervention including medication training, pharmacy monitoring, and medical order review. The control group only has ordinary pharmacists, and the hospital needs to pay the cost of the control group = average pharmacists salary + hospital training pharmacist costs. The intervention group has clinical pharmacist intervention. Assuming that the hourly salary of pharmacist services is equal to the hourly salary of pharmacists, that is, the time cost of intervention provided by the intervention group is calculated by the corresponding theoretical remuneration, then the intervention group cost = average pharmacist salary + time cost + hospital training pharmacist cost.The hospital's training costs for clinical pharmacists in the intervention group consist of specialist clinical pharmacist training costs, annual training costs for clinical pharmacists, and annual licensed pharmacist training costs. General pharmacists in the control group only have annual licensed pharmacist training costs. The study lasted for one year.

Average annual salary of pharmacist = monthly salary of pharmacist×12;

Training fee of control group = the average annual training fee for licensed pharmacists;

Time cost of clinical pharmacists in the intervention group＝The total time of the clinical pharmacist's pharmaceutical intervention × hourly salary;

Intervention group training cost＝Annual cost of specialist training + annual training cost of clinical pharmacist + annual training cost of licensed pharmacist;

The annual cost (AC) calculation formula is ，*i*_0_ is the discount rate, which is 5%, AC is the present value of the cost，*n* is the number of years.

（2）**Effectiveness(E)**  The effect index is the overall rational rates of PPIs.

（3）**Cost effectiveness ratio (C/E)**  The cost-effectiveness ratio (C/E) is used to express the net cost required to obtain an effect. The smaller the ratio, the lower the cost to achieve the effect.

（4）**Incremental analysis** The incremental cost-effectiveness ratio (ΔC/ΔE) is used to calculate the cost of clinical pharmacists for each additional unit of efficacy in the intervention group than in the control group. In this study, the cost effect ratio of the control group was used as the willing payment threshold, if Δ C/ Δ E was lower than C/E of control group, which indicated that intervention group was more economical than control group.

（5）**Sensitivity analysis** Some variables in pharmacoeconomics research are usually difficult to measure accurately, and the uncertainty of data may bias the analysis results. The purpose of sensitivity analysis is to verify that the changes of the analyzed data within a certain limit do not affect the analysis conclusion, so it can be considered that the current analysis is credible. With the further deepening of the reform of the medical and health system, the hospital has co The purpose of cost-effectiveness analysis in this study is to provide reference for the calculation of pharmaceutical service cost in medical institutions in China. Therefore, the change in hourly wages for pharmaceutical services has an important impact on the credibility of the cost-effectiveness analysis of this study. The hourly salary of pharmacists involved in the study fluctuated from - 50% to + 50%, and other parameters remained unchanged, so as to check the reliability of the research results.

**Appendix 4 PPI specifications, DDD and unit price involved in this study**

| **Drug** | **Specification /mg** | | | **DDD/mg** | **unit price /yuan** |
| --- | --- | --- | --- | --- | --- |
| **Rabeprazole sodium lyophilized powder** | | 20 | 20 | | 95 |
| **Pantoprazole sodium lyophilized powder** | | 40 | 40 | | 95.74 |
| **Omeprazole injection** | | 40 | 40 | | 77.78 |
| **Esomeprazole Sodium Lyophilized Powder**  **(Nexium)** | | 40 | 40 | | 100.12 |
| **Esmeprazole sodium lyophilized powder**  **(AiSuPing)** | | 20 | 40 | | 40.51 |
| **Lansoprazole lyophilized powder** | | 30 | 30 | | 20.67 |
| **Esomeprazole enteric capsule**  **（LaiMeiShu）** | | 20 | 40 | | 8.2543 |
| **Omeprazole Enteric-coated Tablets** | | 20 | 20 | | 0.4346 |
| **Esomeprazole magnesium enteric coated tablets**  **（Nexium）** | | 20 | 40 | | 8.8229 |
| **Pantoprazole Enteric-Coated Tablets** | | 40 | 40 | | 9.54 |
| **Iprazole enteric coated tablets** | | 5 | 10 | | 13.0567 |
| **Rabeprazole sodium enteric coated tablets** | | 20 | 20 | | 4.1307 |
| **Lansoprazole capsule** | | 30 | 30 | | 8.19 |

（During the study period, there was no change of PPIs, and there was no adjustment in the price of PPIs before and after the intervention.）
